# Comparison Between Arthroscopic and Histological International Cartilage Repair Society Scoring Systems in Porcine Cartilage Repair Model

**DOI:** 10.1177/19476035211069246

**Published:** 2022-01-31

**Authors:** Jani Puhakka, Eve Salonius, Teemu Paatela, Virpi Muhonen, Anna Meller, Anna Vasara, Hannu Kautiainen, Jussi Kosola, Ilkka Kiviranta

**Affiliations:** 1 University of Helsinki, Helsinki, Finland; 2 Helsinki University Hospital, Helsinki, Finland; 3 Kuopio University Hospital, Kuopio, Finland; 4 Kanta-Hämeen keskussairaala, Hameenlinna, Finland

**Keywords:** cartilage repair, ICRS, ICRS II, arthroscopy, histology, animal cartilage repair model, animal model

## Abstract

**Objective:**

The arthroscopic and histological International Cartilage Repair Society (ICRS) scores are designed to evaluate cartilage repair quality. Arthroscopic ICRS score can give a maximum score of 12 and the histological score can give values between 0% and 100% for each of its 14 subscores. This study compares these methods in an animal cartilage repair model. This study hypothesizes that there is a significant correlation between these methods.

**Design:**

A chondral defect was made in the medial femoral condyle of 18 pigs. Five weeks later, 9 pigs were treated with a novel recombinant human type III collagen/polylactide scaffold and 9 were left untreated to heal spontaneously. After 4 months, the medial condyles were evaluated with a simulated arthroscopy using the ICRS scoring system followed by a histological ICRS scoring.

**Results:**

This porcine cartilage repair model produced repaired cartilage tissue ranging from good to poor repair tissue quality. The mean arthroscopic ICRS total score was 6.8 (SD = 2.2). Histological ICRS overall assessment subscore was 38.2 (SD = 31.1) and histological ICRS average points were 60.5 (SD = 19.5). Arthroscopic ICRS compared with histological ICRS average points or its overall assessment subscore showed moderate correlation (r = 0.49 and r = 0.50, respectively). The interrater reliability with the intraclass correlation coefficients for arthroscopic ICRS total scores, histological ICRS overall assessment subscore, and ICRS average points showed moderate to excellent reliability.

**Conclusions:**

Arthroscopic and histological ICRS scoring methods for repaired articular cartilage show a moderate correlation in the animal cartilage repair model.

## Introduction

Articular cartilage is a highly specialized connective tissue present in diarthrodial joints, allowing skeletal load transfer during motion due to its sophisticated composition. Articular cartilage consists of chondrocytes embedded in a hydrated extracellular matrix (ECM), including mainly collagen fibers and proteoglycans. This organized structure of ECM enables the unique viscoelastic and mechanical properties of hyaline cartilage.

Injuries and degeneration of the joint cause changes in the structure of hyaline cartilage, leading to abnormal joint function as well as pain and disability. To relieve these symptoms, cartilage repair aims to fill a defect with repair tissue that has a similar structure and biomechanical function as the original articular cartilage.^1,2^ Hyaline cartilage lacks blood vessels and lymphatic supply, making repair of cartilage lesions challenging.^3^ Surgical therapies ranging from bone marrow stimulation to osteochondral grafting and autologous chondrocyte implantation have been used to overcome this challenge.^4,5^ Along with developing these cartilage repair techniques, assessing the result of these repair procedures has become more critical. Various methods, including imaging techniques,^6^ arthroscopy,^7,8^ and histology,^9^ have been used to assess the tissue morphology to evaluate cartilage repair success.

As the higher quality of repair tissue correlates with better clinical outcomes, repair quality may be an objective measure to evaluate the repair technique.^10-12^ With arthroscopic and histological methods, cartilage pathologies and cartilage repair’s structural outcome may be evaluated.^1,13-17^ In addition, repair tissue quality may serve as a primary outcome measure in studies without achievable clinical outcomes (e.g., in feasibility studies and animal studies).^7^

Histological evaluation of articular cartilage gives a detailed information of the tissue structure. However, it is an invasive method requiring a cartilage biopsy that might cause additional tissue morbidity. In a clinical setting, an arthroscopic evaluation system that does not require a tissue biopsy and correlates with histological findings could diminish the need for more invasive methods to assess cartilage repair results.

The International Cartilage Repair Society (ICRS) score and the Oswestry Arthroscopy Score (OAS) have often been used to evaluate the quality of cartilage repair arthroscopically.^7,10^ Goebel *et al*.^18^ have published a scoring system to evaluate articular cartilage repair macroscopically. The Modified O’Driscoll Scale (MODS) and ICRS gradings systems have been used for histological evaluation of cartilage repair.^19-21^

In 2003, an arthroscopic ICRS scoring system was published.^10^ The arthroscopic ICRS grading system has been validated to assess repaired articular cartilage.^7,8^ It consists of the subscores producing a total score (**Table 1**).

**Table 1.** Arthroscopic Evaluation of Cartilage Repair With ICRS Scoring System.^10^

Cartilage Repair Assessment ICRS	Points
Degree of defect repair	
In level with surrounding cartilage	4
75% repair of defect depth	3
50% repair of defect depth	2
25% repair of defect depth	1
0% repair of defect depth	0
Integration to the border zone	
Complete integration with surrounding cartilage	4
Demarcating border < 1 mm	3
3/4th of graft integrated, 1/4th with a notable border > 1 mm width	2
1/2 of graft integrated with surrounding cartilage, 1/2 with a notable border > 1 mm	1
From no contact to 1/4th of graft integrated with surrounding cartilage	0
Macroscopic appearance	
Intact smooth surface	4
Fibrillated surface	3
Small, scattered fissures or cracks	2
Several, small or few but large fissures	1
Total degeneration of the grafted area	0
Overall repair assessment	
Grade I: normal	12
Grade II: nearly normal	11-8
Grade III: abnormal	7-4
Grade IV: severely abnormal	3-1

ICRS = International Cartilage Repair Society.

The Histological Endpoint Committee of the ICRS developed a histological scoring system to evaluate the quality of repair tissue in 2003.^21^ This scoring system, called the ICRS Visual Assessment Scale, consists of 6 subscores: matrix, cell distribution, subchondral bone, surface, cartilage mineralization, and cell population viability. While studying the reliability of this scoring system, Mainil-Varlet and co-workers developed an improved histological scoring method called the ICRS II in 2010.^20^ The ICRS II score contains 14 subscores, each evaluated on a 100 mm visual analog scale (VAS; **Table 2**). The reliability of ICRS II was found to be better compared with earlier scoring systems.^20^ The subscores “overall assessment” and “matrix staining” have shown the highest correlation coefficients for interreader and intrareader variability.^20^ Since then, the ICRS II scoring system has been used as an outcome measure in cartilage repair studies.^16,17,22,23^

**Table 2.** ICRS II Parameters.^20^

Histological Parameter	Score
1. Tissue morphology (viewed under polarized light)	0%: Full-thickness collagen fibers 100%: Normal cartilage birefringence
2. Matrix staining (metachromasia)	0%: No staining 100%: Full metachromasia
3. Cell morphology	0%: No round/oval cells 100%: Mostly round/oval cells
4. Chondrocyte clustering (4 or more grouped cells)	0%: Present 100%: Absent
5. Surface architecture	0%: Delamination, or major irregularity 100%: Smooth surface
6. Basal integration	0%: No integration 100%: Complete integration
7. Formation of a tidemark	0%: No calcification front 100%: Tidemark
8. Subchondral bone abnormalities/marrow fibrosis	0%: Abnormal 100%: Normal marrow
9. Inflammation	0%: Present 100%: Absent
10. Abnormal calcification/ossification	0%: Present 100%: Absent
11. Vascularization (within the repaired tissue)	0%: Present 100%: Absent
12. Surface/superficial assessment	0%: Total loss or complete disruption 100%: Resembles intact articular cartilage
13. Mid/deep zone assessment	0%: Fibrous tissue 100%: Normal hyaline cartilage
14. Overall assessment	0%: Bad (fibrous tissue) 100%: Good (hyaline cartilage)

ICRS = International Cartilage Repair Society.

The relationship between these 2 scoring methods assessing the results of cartilage repair surgery is unknown. Therefore, we aimed to evaluate the correlations between arthroscopic and histological ICRS scoring methods with a hypothesis that there should be a significant correlation between these 2 methods used for evaluating cartilage repair tissue quality.

## Materials and Methods

### Experimental Animals and Ethical Considerations

Four-month-old domestic pigs (*Sus scrofa domestica*, *n* = 18), obtained from a local farmer, were used for this study. The animals were acclimatized to the experimental facility and handlers for 14 days before any treatment. The animals were housed in groups and allowed free movement in pens with bedding throughout the experiment. A veterinarian supervised the well-being of the animals during the study. The study was authorized by the National Animal Experiment Board (ESAVI/6113/04.10.07/2015) and conducted according to the ethical guidelines and regulations of the Finnish Act on the Protection of Animals Used for Scientific or Educational Purposes (497/2013) and Government on the Protection of Animals Used for Scientific or Educational Purposes (564/2013). The same experimental animals were also used in a different study.^24^

### Surgical Procedure

For the initial procedure, animals were anesthetized with 0.2 mg/kg medetomidine, 10 mg/kg ketamine, and 3 mg/kg propofol, followed by 1.5% to 2.5% isoflurane. Preoperative analgesia of 0.05 mg/kg buprenorphine and 3 mg/kg carprofen, as well as antibiotic prophylaxis of 3.0 g cefuroxime, was administered. The animals were intubated and set in a supine position on the operating table. A medial parapatellar arthrotomy was made to the right hind leg and the patella was dislocated laterally. Initially, a full-thickness oval-shaped chondral defect was made in the right medial femoral condyle of all 18 pigs. The defects were made with a specially designed instrument (size 11 × 17 mm, area 1.5 cm^2^) to standardize the defect size. The defect size was selected with a pilot study seeking the largest possible defect that would still allow suturing of the scaffold to its outer rim. The subchondral bone at the defect area was left intact so that the bone surface just started bleeding. The animals were allowed free weightbearing and unrestricted movement after the operation. Postoperative analgesia with carprofen and buprenorphine together with microbiological prophylaxis of cephalexin was continued for 3 days.

Five weeks later, using the described anesthesia protocol, each of the 18 pigs’ defect area was debrided and 9 of those pigs were treated with a novel recombinant human type III collagen/polylactide scaffold.^25,26^ Nine pigs did not receive the scaffold after the debridement and the defect was left to heal spontaneously. All surgical procedures were made by 2 (A.V. and T.P.) experienced orthopedic knee surgeons.

### Simulated Arthroscopic Evaluation

Four months after the second procedure, the pigs were sacrificed with intravenous anesthetic and the medial condyles were excised and evaluated in a simulated video-recorded arthroscopy. Simulated arthroscopy was chosen because the simulated setting was considered to overcome the technical difficulties in small pig’s stifle joint and to assure that no iatrogenic damage was done to the studied cartilage impairing the histological results.

**Figure 1.**
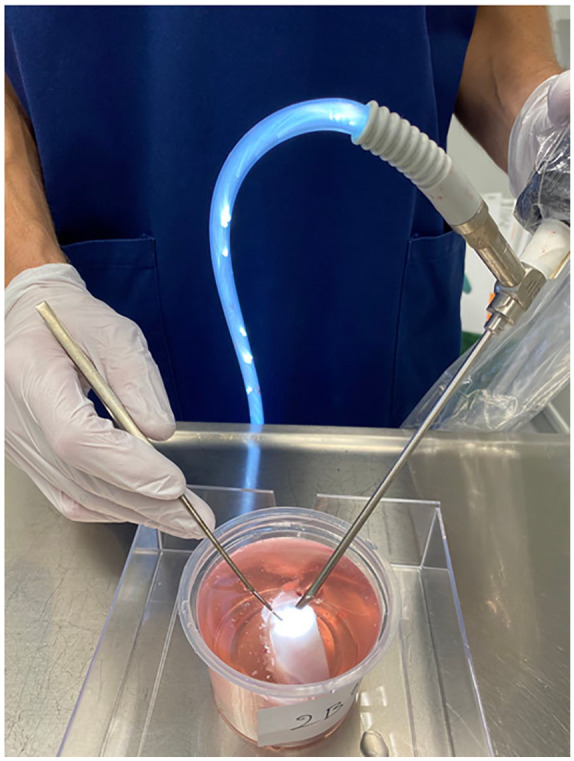
Simulated video-recorded arthroscopy with a camera and a probe.

The simulation was done by immersing the specimens in a container with size 8 × 12 cm filled with phosphate-buffered saline containing metalloprotease inhibitors 5 mM EDTA (ethylenediaminetetraacetic acid disodium salt, VWR International, Fontenay, France) and 5 mM benzamidine hydrochloride (Sigma-Aldrich, St. Louis, MO, USA; PBSI; **Fig. 1**). The excised medial condyles were fixed at the bottom of the container and arthroscopy was performed and video recorded using a standard arthroscopy tower (Karl Storz Endoscopy, Tuttlingen, Germany) with a standard 4.0 mm and 30° angled optic and a standard arthroscopic probe (**Fig. 1**).

The simulated arthroscopy of the repaired tissue was made by 2 surgeons using the ICRS scoring method. The surgeons made their evaluations blinded from the treatment group and the results of the other colleague. The surgeons had different levels of experience with arthroscopic knee procedures. The first evaluator (E.S.) had more than 1 year of experience in knee arthroscopies. The second evaluator (J.P.) had 6 years of experience in knee arthroscopies. Both evaluators were familiar with the ICRS scoring method, but they were also reeducated with the grading system before the initial arthroscopic assessments. During the arthroscopies, the evaluator filled a blank ICRS formula with all ICRS subclasses (**Table 1**).

### Histology

The collected condyle specimens were cut around the defect area and halved from the middle of the defect with a diamond saw. The samples were fixed with 10% buffered formalin for 4 weeks, followed by decalcification in a solution containing 10% EDTA, 4% formalin, and 0.1M phosphate buffer. The samples were embedded in paraffin and the samples containing the lateral half of the defect were cut to 5 µm sections and stained with Safranin-O for histological analyses. Safranin-O-stained sections were blinded and evaluated by the same 2 surgeons who made the arthroscopic evaluation (E.S. and J.P.) using the ICRS II score.^20^ Both surgeons had been educated and had earlier experience performing histopathological evaluations as basic science researchers.

### Statistics

In arthroscopic ICRS grading system, the assessment is made from the sum of the points from the subclasses. The histological ICRS scoring method has 14 subclasses. Subclass Overall assessment is a subjective rating from 0 to 100 using the VAS (**Table 1**) and has shown to have good interrater reliability^20^ and thus chosen for comparison together with the arithmetic average from all the 14 subclasses. Arthroscopic and histological ICRS scores were transformed to normality using rank-based normalization method by van der Waerden.^27^ Correlation coefficients between the arthroscopic ICRS total points and histological ICRS average points or overall assessment subscore with 95% confidence interval (CI) were calculated using Spearman correlation using Sidak adjusted probabilities.

Two reliability measures were used to evaluate the interrater reliability of used scoring systems. Intraclass correlation coefficients (ICCs) were calculated using a 2-way random-effects model with single rater type with absolute agreement. The ICC values less than 0.5 indicate low reliability, between 0.5 and 0.75 moderate reliability, between 0.75 and 0.9 good reliability, and greater than 0.90 excellent reliability.^28,29^ Internal consistency was estimated using Cronbach α coefficient with an α value of 0.7 to 0.8 being interpreted as satisfactory; for clinical application, the α value should be 0.9 or more.^30^

All statistical analyses were performed with Stata version 16.0 (StataCorp, College Station, TX, USA).

## Results

**Figure 2.**
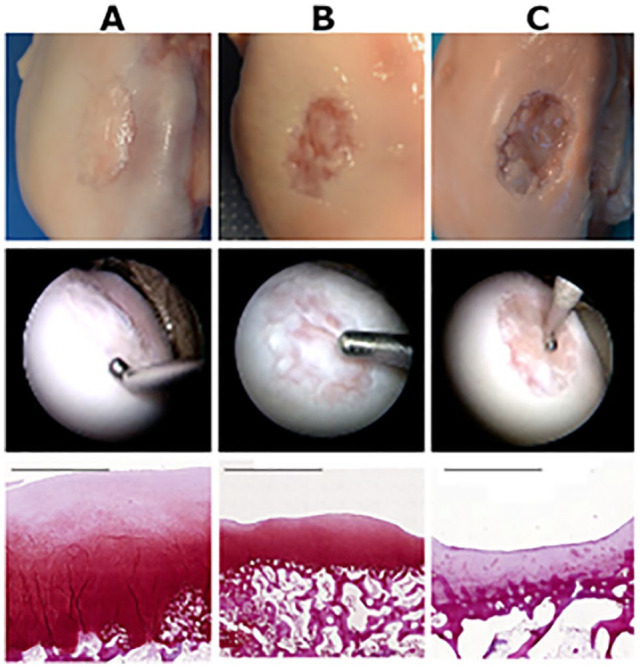
Examples of macroscopic, arthroscopic, and histological cartilage repair results: (**A**) Good quality repair: good quality repair tissue with good defect fill, (**B**) average quality repair: some regenerated cartilage, (**C**) poor quality repair: little regenerated cartilage. Selecting examples to be poor, average, and good was made using the macroscopic Göbel scoring method.^18^ Scale bar: 2 mm.

The porcine cartilage repair model produced repair tissue from poor to good quality (**Fig. 2**). According to arthroscopic ICRS overall repair assessment (**Table 1**), repair tissue quality was severely abnormal (grade IV) in 1 animal, abnormal (grade III) in 7 animals, and nearly normal (grade II) in 10 animals.

An arthroscopic evaluation was performed to all 18 pigs, but due to technical error in histological staining procedure, 1 pig was excluded from the histological evaluation.

The internal consistency (Cronbach α) for the ICRS items was 0.862 in the arthroscopy, 0.955 for the histological ICRS II overall assessment subscore, and 0.967 for the ICRS II average points.

The interrater reliability with the ICC for arthroscopic ICRS total scores, histological ICRS II overall assessment subscore, and ICRS II average points showed moderate to excellent reliability (**Table 3**).

**Table 3.** Interrater Reliability Evaluated Repaired Cartilage With ICRS Arthroscopic Scoring System and ICRS Histological Scoring System (ICRS II).

Scoring System	ICC (95% CI)
ICRS arthroscopic scoring system	0.738 (0.432-0.893)
ICRS II overall assessment subscore	0.918 (0.789-0.969)
ICRS II average points	0.938 (0.839-0.977)

Represented are ICC estimates and 95% CI based on an absolute agreement, a 2-way random-effects model. ICRS = International Cartilage Repair Society; ICC = Intraclass correlation coefficients; CI = confidence interval.

Average scores for arthroscopic and histological assessments are shown in **Table 4**.

**Table 4.** Average Scores With Standard Deviation for Arthroscopic and Histologic (ICRS II Overall Assessment Subscore/Average Points).

Scoring System	Mean (SD)
ICRS Arthroscopic total score	6.8 (2.2)
ICRS II overall assessment subscore	38.2 (31.1)
ICRS II average points	60.5 (19.5)

ICRS = International Cartilage Repair Society.

**Figure 3.**
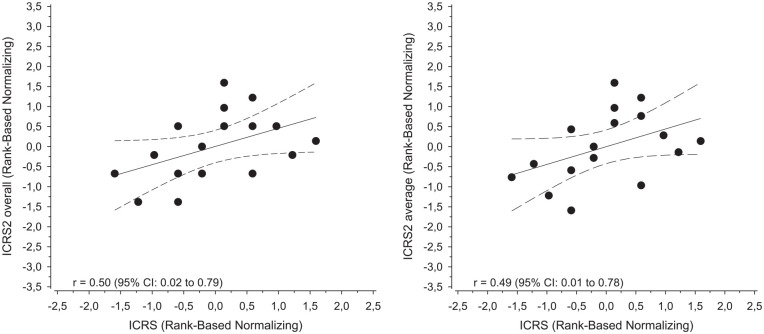
Correlation plot and regression line with 95% confidence interval (dashed lines) comparing arthroscopic ICRS score with histological ICRS II overall assessment subscore and the average of all ICRS II subscores after normalization. ICRS = International Cartilage Repair Society.

Arthroscopic ICRS compared with histological ICRS II overall assessment subscore or average points showed moderate correlation **Figure 3**.

## Discussion

This study design produced a broad spectrum of repair results from poor to good repair tissue quality to test the correlation between the arthroscopic and histological evaluation of the cartilage repair results in an animal cartilage repair model. To our knowledge, this is the first study to compare arthroscopic and histological ICRS cartilage repair scoring methods. This study showed a moderate correlation between arthroscopic ICRS score compared with histological ICRS II overall assessment subscore or average points.

This study was not specifically designed to analyze the reliability of these scoring methods. A reliability analysis was made to see whether the 2 observers agreed to the same degree as the earlier reliability studies have suggested and by that making the correlation analysis feasible. For arthroscopic ICRS score, Smith *et al*.^7^ reported the interrater reliability to be 0.83 using the ICC. In the study by van den Borne *et al.*,^8^ the interrater reliability was 0.62. In this study, the interrater reliability was similar to the earlier studies showing moderate reliability for arthroscopic ICRS scores (ICC = 0.738).

For histological ICRS II scoring, Mainil-Varlet *et al.*^20^ reported good interrater reliability for the overall and matrix staining subscore. We studied the interrater reliability for histological ICRS II overall assessment subscore and the average of all subscores showing excellent interrater reliability for both (ICC = 0.918 and ICC = 0.938, respectively). However, the results could not be compared due to the lack of reporting the statistical method used in the Mainil-Varlet *et al.*^20^ study.

The interrater reliability was better for the histological ICRS II scoring system than for the arthroscopic ICRS cartilage repair scoring system. This might be because arthroscopy gives more visual and tactile information by allowing evaluation to be made from different angles using a probe. On the contrary, the histological ICRS assessment is done from a preselected single stained section showing a more detailed structure but only the selected section.

In this study, with a 1.5 cm^2^ defect area, the repair tissue showed uneven quality within the defect area. It was typical in both intervention groups that the more weightbearing lateral edge of the large defect presented good filling, but the medial half manifested with a poor repair. A mechanical stimulus is critical for cartilage development and homeostasis^2^; it improves chondrocyte viability^31^ and cartilage extracellular matrix deposition^32^ *in vivo* and *ex vivo*. The lateral portion of the medial condyle bears weight^33^ and might therefore have an improved cartilage repair capacity. As cartilage repair tissue often has heterogeneous quality, the arthroscopic evaluation has more dimensions to interpret because the whole repair area is visible. Heterogenous repair tissue might make arthroscopic evaluation less reliable. Better reliability does not necessarily make histological evaluation a better tool for evaluating cartilage repair results as the repair tissue quality differs much within a repaired defect. Histological evaluation interprets the quality of repair tissue only from the selected section disregarding the other repaired areas.

The present study’s limitations are that we had only 2 raters and the sample size was relatively small. The 2 raters also had different experiences in arthroscopic skills, which may affect the arthroscopic evaluation of the cartilage repair. However, in our earlier study regarding the reliability of arthroscopic scoring, the intrarater or interrater reliability was not affected by the experience on the evaluator.^24^ In addition, the repair tissue was evaluated with arthroscopy in a simulated setup and not in the joint, which may impair the results’ generalization. In the present study, a simulated arthroscopy setting was used because the pig’s stifle joint is small and the experience of a surgeon might affect the diagnostic accuracy of the pig’s stifle joint arthroscopy.^34^ Therefore, the simulated setting was considered to minimize technical difficulties and provide equal visualization of the repair site for both observers with different arthroscopic skills. A simulated arthroscopy can also be seen as a benefit because it made the arthroscopic evaluation possible for more than one surgeon and assured that no iatrogenic damage was done to the studied cartilage. Simulated arthroscopy differs from macroscopic evaluation. With simulated arthroscopy, the lesion can be evaluated with particular magnification from different angles mimicking a real arthroscopic cartilage evaluation in a joint.

The lesion size and the repair quality can affect the reliability of an evaluation method. Arthroscopic ICRS score is more reliable for small repaired lesions with good repair quality than for extensive lesions.^35^ The strength of this study is a study design producing a wide variety of repair results with scaffold-based repair techniques and a repair solely relying on spontaneous healing with the initial cartilage lesion performed with not only a removal of the cartilage but also the calcified cartilage layer until it just started bleeding. This may have further supported the tissue repair with introduced cells from surrounding tissues and circulation. The repaired lesions were standardized to a fixed size, minimizing small repaired defects’ mixing effect versus large repaired defects. Also, the observers were the same for both arthroscopic and histological evaluations.

Histological repair assessment is seen as a reliable and objective measure of repair quality and the treatment’s success.^20^ The results of this study indicate that the ICRS arthroscopic score is a valuable tool for evaluating articular cartilage repair showing a moderate correlation with histological ICRS II scores. While the histological ICRS II score better shows the repair tissue’s structural quality, it only represents cartilage repair from a single section in the heterogeneous repair tissue area. An arthroscopic evaluation covers the whole repair result but lacks in reliability, according to our findings. Both scoring methods lack the inherent capability to reliably describe the heterogeneous repair results of a large chondral defect. Knowing the correlation between arthroscopic and histological scoring has a clinical value. Furthermore, it is essential to know to what extent the arthroscopic findings correlate to histology as the novel methods for cartilage repair rely on restoring the tissue at the cellular level as close to the healthy native tissue as possible. Unfortunately, biopsy and histological assessments are often not feasible in the clinical setting.

New objective methods to assess the severity of damage in hyaline cartilage during arthroscopy have been introduced, for example, high-frequency ultrasound,^36,37^ mechanical testing of cartilage stiffness,^38,39^ mechano-acoustical testing,^40,41^ optical coherence tomography^42^ and electromechanical testing.^43-45^ These methods may have the ability to make an arthroscopic assessment of cartilage repair more reliable and accurate. Still, none of them has been validated to assess repair tissue quality. Their validity has been studied to evaluate the damage of native hyaline cartilage. It is unknown whether an excellent ability to detect damage in hyaline cartilage also makes it a reliable and useful instrument to evaluate cartilage repair quality.

## Conclusions

Based on the present study, arthroscopic and histological ICRS scoring methods for repaired articular cartilage show a moderate correlation in the animal cartilage repair model.

## Research Data

sj-xlsx-1-car-10.1177_19476035211069246 – Supplemental material for Comparison Between Arthroscopic and Histological International Cartilage Repair Society Scoring Systems in Porcine Cartilage Repair Model Click here for additional data file. Supplemental material, sj-xlsx-1-car-10.1177_19476035211069246 for Comparison Between Arthroscopic and Histological International Cartilage Repair Society Scoring Systems in Porcine Cartilage Repair Model by Jani Puhakka, Eve Salonius, Teemu Paatela, Virpi Muhonen, Anna Meller, Anna Vasara, Hannu Kautiainen, Jussi Kosola and Ilkka Kiviranta in CARTILAGE This article is distributed under the terms of the Creative Commons Attribution 4.0 License ( https://creativecommons.org/licenses/by/4.0/) which permits any use, reproduction and distribution of the work without further permission provided the original work is attributed as specified on the SAGE and Open Access pages ( https://us.sagepub.com/en-us/nam/open-access-at-sage).
